# Inflammation Induces TDP-43 Mislocalization and Aggregation

**DOI:** 10.1371/journal.pone.0140248

**Published:** 2015-10-07

**Authors:** Ana Sofia Correia, Priyanka Patel, Kallol Dutta, Jean-Pierre Julien

**Affiliations:** 1 Centre de Recherche du Centre Hospitalier Universitaire de Québec, 2705 Boulevard Laurier, Québec, QC, G1V 4G2, Canada; 2 Research Centre of Institut universitaire en santé mentale de Québec, Department of Psychiatry and Neuroscience, Laval University, Québec, QC, Canada; Department of Pathology, Anatomy & Cell Biology, Thomas Jefferson University, UNITED STATES

## Abstract

TAR DNA-binding protein 43 (TDP-43) is a major component in aggregates of ubiquitinated proteins in amyotrophic lateral sclerosis (ALS) and frontotemporal lobar degeneration (FTLD). Here we report that lipopolysaccharide (LPS)-induced inflammation can promote TDP-43 mislocalization and aggregation. In culture, microglia and astrocytes exhibited TDP-43 mislocalization after exposure to LPS. Likewise, treatment of the motoneuron-like NSC-34 cells with TNF-alpha (TNF-α) increased the cytoplasmic levels of TDP-43. In addition, the chronic intraperitoneal injection of LPS at a dose of 1mg/kg in TDP-43^A315T^ transgenic mice exacerbated the pathological TDP-43 accumulation in the cytoplasm of spinal motor neurons and it enhanced the levels of TDP-43 aggregation. These results suggest that inflammation may contribute to development or exacerbation of TDP-43 proteinopathies in neurodegenerative disorders.

## Introduction

Amyotrophic lateral sclerosis (ALS) is a neurodegenerative disorder characterized by the loss of motor neurons in the brain and spinal cord, causing progressive muscle weakness and typically leading to death by paralysis within a few years. Mutations in over twenty genes are known to be associated with familial forms of ALS [1–2] which account for 10% of all ALS cases. In both familial and sporadic ALS, degenerating neurons are known to present an abnormal accumulation of cytoplasmic inclusions containing ubiquitinated proteins [3]. TAR DNA-binding protein (TDP-43) has been identified as a major component of cytoplasmic inclusions in sporadic and most familial ALS cases, as well as in frontotemporal lobar dementia (FTLD) with ubiquitinated inclusions, coupling these two diseases as TDP-43 proteinopathies [4–9]. Various dominant mutations in TDP-43 have also been linked with familial cases of both ALS and FTLD, confirming the importance of TDP-43 in the pathology of these diseases [10–16].

Under normal conditions TDP-43 is mostly localized in the nucleus, where it is mainly involved in RNA processing [17–19]. In degenerating neurons of patients with ALS and FTLD, TDP-43 accumulates in the cytoplasm and forms insoluble aggregates in the nucleus, cytoplasm or processes [4, 7]. Aberrant cytoplasmic TDP-43 is known to be truncated into C-terminal fragments (CTFs), phosphorylated and/or ubiquitinated [9, 7, 20]. The cellular pathways causing TDP-43 proteinopathy are not fully elucidated albeit some factors are known to induce TDP-43 mislocalization in the cytoplasm including axotomy, cell stress, TDP-43 gene mutations and overexpression [17, 21, 22].

Previously, we reported that levels of messenger RNA (mRNA) and protein for TDP-43 and nuclear factor κ B (NF-κB) p65 were higher in the spinal cord of ALS patients than of control individuals [23]. Surprisingly, TDP-43 was found to interact with NF-κB p65 in glia and neurons of ALS patients and of transgenic mice overexpressing human wild-type or mutant TDP-43 species. NF-κB is a key component of the innate immune response. This led us to investigate the potential effects of NF-κB activation by inflammatory stimuli on TDP-43 redistribution in various cultured cells including microglia, astrocytes and neurons. It is well established that dysfunction glial cells can contribute to motor neuron damage [24–26]. Moreover, it is noteworthy that ALS patients exhibit increased levels of lipopolysaccharides (LPS) in the blood as well as an up-regulation of LPS/TLR-4 signaling associated genes in peripheral blood monocytes [27–28].

Here, we report that LPS exposure induced cytoplasmic redistribution of TDP-43 in cultured microglia and astrocytes. Similarly, NF-κB activation in motor neuron-like cell line NSC-34 by TNF-α enhanced TDP-43 cytoplasmic level. We also tested the *in vivo* effect of chronic LPS administration in transgenic mice expressing genomic fragment of human TDP-43 A315T gene (hTDP-43^A315T^) [11–12]. Interestingly, the chronic LPS treatment enhanced the cytoplasmic mislocalization and aggregation of TDP-43 in the spinal cord of TDP-43 ^A315T^ transgenic mice. These results suggest that chronic brain inflammation may contribute to TDP-43 proteinopathies.

## Materials and Methods

### Animals used

The heterozygous transgenic mouse line expressing the human mutant TDP-43^A315T^ (hTDP-43^A315T^) has been generated and characterized by us [29, 23]. All experimental procedures were approved by the Laval University Animal Care Ethics Committee and are in accordance with the Guide to the Care and Use of Experimental Animals of the Canadian Council on Animal Care.

### Astroglia cultures

Primary astroglial cultures from brain tissues of neonatal (P2-P3) mice were prepared as described previously [30]. In brief, the brain tissues were stripped of their meninges and minced with scissors under a dissecting microscope in Dulbecco’s modified Eagle medium (DMEM). After trypsinization (0.25% trypsin-EDTA (Life Technologies), 10 min, 37°C, 5% CO_2_), the tissue was triturated. The cell suspension was washed in glial culture medium (DME supplemented with 10% FBS, 1 mM l-glutamine, 1 mM Na pyruvate, 100 U/ml penicillin, and 100 mg/ml streptomycin, non-essential amino acids (all from Life Technologies) and cultured at 37°C, 5% CO_2_ in 25 cm^2^ Falcon tissue culture flasks (BD, one brain per flask) coated with 10 mg/ml poly-d-lysine (PDL; Sigma-Aldrich) for overnight and then rinsed thoroughly with sterile distilled water. Four to five days later medium was changed and supplemented with 5ng/ml of mouse recombinant macrophage colony stimulating factor (M-CSF, R&D) and every second day thereafter, for a total culture time of 10–14 days. At the moment of tissue dissection the genotype of each pup was unknown. Each brain was then dissected and cultured separately (one brain/25 cm^2^ flask). The transgenic pups were identified by DNA extraction from tails and PCR amplification of the human *TARDBP* gene. After 10–14 days, when the genotype of each pup was known, cultures with the same genotype were pulled together during the separation of astrocytes from microglia.

### Separation of astrocytes from microglia

The procedure followed to separate astrocytes from microglia is based in previous published protocols [30]. In brief, microglia were shaken off the primary mixed brain glial cell cultures in an orbital shaker set at 200 rpm, 37°C, for 3h. Microglia cells in suspension were collected and re-plated for expansion in glial culture medium supplemented with M-CSF and that had been conditioned on astrocyte cultures. Medium in the microglia cultures was changed every 5–7 days. Cells that remained attached after the orbital shaking, which were mainly a monolayer of astrocytes, were dissociated with 0.5% Trypsin-EDTA diluted in DMEM ratio 1:3 for 15 min at 37°C 5%CO2. In order to eliminate microglia cells contaminating the monolayer of astrocytes, dissociated cells were re-plated and microglia cells were let to attach to the surface of the culture flask for 1h, while astrocytes were still in suspension. Astrocytes were then collected and re-plated with glial culture medium. Medium in astrocytes cultures was changed every 2–3 days. Medium collected from astrocyte cultures were filtered through 0.2 um filters (Millipore) and kept at 4°C. Astrocyte-conditioned glial culture medium was used to maintain the microglia cultures. After expanded for 10–14 days, microglia were re-plated at a density of 20,000 cell/cm^2^ and astrocytes at 40,000 cell/cm^2^ in 16-well chamber slides (Thermo scientific) for immunocytochemical analysis and 6-well-plates for protein or RNA extraction. Astrocytes and microglia cultures were treated with LPS (Sigma) at different concentration and for different periods as indicated in figures and results section.

### Intraperitoneal LPS injection in mice

To trigger a systemic innate immune response in the CNS, presymptomatic 6-month-old hTDP-43^A315T^ mice and their non-transgenic (wild-type) littermates received intraperitoneal (i.p.) injection of LPS (1 mg/kg of body weight; from Escherichia coli; serotype 055:B5; Sigma, Saint Louis, MO) diluted in 100 μl of vehicle (Veh) solution (sterile pyrogen-free saline). Mice were i.p. injected once a week for duration of two months. Mice were not exhibiting overt phenotypes due to LPS injection. Control mice were given same volume of saline.

### Preparation of spinal cord sections for immunohistochemistry

After two months of systemic injections, animals were deeply anesthetized by i.p. injection of pentobarbital (50mg/kg) and then rapidly perfused transcardially with 0.9% saline, followed by 4% paraformaldehyde in 0.1 M borax buffer, pH 9.5, at 4°C. Spinal cords were rapidly removed from the animals. Dissected spinal cord tissues were postfixed for 24 h in 4% paraformaldehyde and equilibrated in a solution of PBS-sucrose (20%) for 48 h. Spinal cord tissues were cut in 25 μm thick sections with a Leica frozen microtome and kept in a cryoprotective solution at -20°C. Immunohistochemistry was performed on 25μm-thick sections. TDP-43 was probed (1:200) with Rabbit polyclonal (ProteinTech) or (1:200) mouse monoclonal (Abnova) antibodies. Neu N was immunostained with (1: 500) monoclonal (Invitrogen) antibody.

### Quantitative real-time PCR

Real-time RT-PCR was performed with a LightCycler 480 (Roche) sequence detection system using Light-Cycler SYBR green I at the Quebec Genomics Centre. Total RNA was extracted from cell cultures using TRIZOL reagent (Life Technologies). Total RNA was treated with DNase (QIAGEN) to get rid of genomic DNA contaminations. Total RNA was then quantified using Nanodrop, and its purity was verified by Bioanalyzer 2100 (Agilent Technologies). Gene-specific primers were constructed using the GeneTools software (Biotools Inc.). Three genes, Atp5, Hprt1, and GAPDH, were used as internal control genes. The mRNA copy number of each analyzed gene was divided by the mRNA copy number of the house-keeping gene Atp5 in the same sample, in order to take in account variations in samples size. Then, in order to get the fold changes in the expression of each gene with the LPS-treatment, levels of mRNA in the LPS-treated sample were divided by the levels of mRNA in the respective untreated control. Fold change equal to 1 indicate no effect of LPS in the gene expression, while fold changes higher than 1 indicate that the LPS treatment led to a higher expression of the analyzed gene.

### Sub-cellular fractionation

For sub-cellular fractionation, cells were lysed by a hypotonic buffer (10 mM HEPES-KOH pH 7.6, 10 mM KCl, 1.5 mM MgCl2, 1 mM EDTA, 1 mM EGTA, 0.5 mM DTT, Halt phosphatase inhibitor cocktail (Thermo Scientific) and protease inhibitor (Sigma)) for 30 min on ice. Cells were further broken down by passing 30 times though a 22.5/27 G needle. Membranes were separated from the remaining cellular components by centrifugation at 3000 rpm, 10 min., 4°C. Pellet was then resuspended in extraction buffer (20 mM HEPES-KOH pH 7.6, 25% v/v glycerol, 0.5 mM NaCl, 1.5 mM MgCl2, 1 mM EDTA, 1 mM EGTA, 0.5 mM DTT, Halt phosphatase inhibitor cocktail (Thermo Scientific) and protease inhibitor (Sigma)) and incubated for 60 min at 4°C with rotation. Nuclear fraction was then separated from the cytosolic fraction by ultra centrifugation at 30,000g for 30 min at 4°C. Pellet containing the cytosolic fraction was resuspended in RIPA buffer. Protein concentration was estimated using the Bradford method.

### Soluble and Insoluble fractionation

It was done as previously described by Hart et al, with some modifications as described below [31]. Frozen spinal cords of LPS or saline-injected mice were homogenized in ice-cold homogenisation buffer (NP40 lysis buffer) containing 20 mM Tris-HCl, PH 7.4, 150mM NaCL, 1% NP40, 5mM EDTA, 1 mM DTT, 10% glycerol, 1 mM EGTA), freshly supplemented with protease inhibitor cocktail (Sigma) and phosphatase inhibitors (10 mM NaF, 1 mM b-glycerophosphate, 1 mM Na3VO4). Lysates were rotated for 30 minutes at 4°C and then centrifuged at 4°C for 20 minutes at 15,800 g. Supernatants containing salt-soluble fraction were transferred to new tubes. To remove carryovers the pellet was washed once in homogenisation buffer and resuspended in Urea buffer (homogenisation buffer with 8 M urea, supplemented with protease and phosphatase inhibitor) followed by sonication. After spinning the lysate at 4°C for 20 minutes, the supernatant was removed as insoluble fraction. Proteins were quantified by Lowry method.

### Western blots

Total protein was extracted from cells using the RIPA buffer (50 mM TRIS-HCl pH7.4, 1 mM EDTA pH 8.0, 150 mM NaCl, 0,1%SDS, 1% NP-40, Halt phosphatase inhibitor cocktail (Thermo Scientific) and protease inhibitor (Sigma)). Protein concentration was estimated using the Bradford method. After denaturation, protein samples were resolved by 12% sodium dodecyl sulphate polyacrylamide gel electrophoresis (SDS-PAGE) and transferred onto nitrocellulose membrane (Schleicher & Schuell, Dassel, Germany). Membranes were incubated in blocking solution (5% milk, 0.1% Tween-20 in Tris-buffered saline (TBS) solution) for 1h at room temperature. Membranes were then incubated with a primary antibody diluted in blocking solution at 4°C overnight. After rinsing 3 times with 0.1% Tween-20 in TBS solution, membranes were incubated in a horseradish peroxidase-conjugated secondary antibody made in goat (Jackson ImmunoResearch Laboratories Inc., Baltimore, PA, USA) diluted 1:5000 in blocking solution. After rinsing 3 times with 0.1% Tween-20 in TBS solution, immunoreactive proteins were visualized by chemiluminescence with the Renaissance kit (PerkinElmer Life Sciences, Waltham, MA, USA). Primary antibodies used were the following: (1) Anti human TDP-43 monoclonal antibody (1: 1000, Abnova) (2) anti-TDP43 rabbit polyclonal antibody (1:4000, ProteinTech #10782-2-AP), which reacts with both human (transgenic) and mouse (endogenous) proteins and (3) anti-actin mouse monoclonal (1:25000, Chemicon). Bands intensity was measured using Image J 1.45p (NIH, USA). The intensity of all TDP-43 bands was divided by the intensity of the respective actin band to take in account the differences in the protein loading. Fold change is the ratio of the band intensity in the LPS-treated cultures over the band intensity in the respective untreated controls, after normalized by the intensity of the correspondent actin bands.

### Immunofluorescence, microscopy and image processing

Paraformaldehyde (4%) fixed cell cultures and 20-um-thick spinal cord sections were incubated in blocking solution (10% normal goat serum, 0.25% Triton-X100 in phosphate buffered saline (PBS) for 1h at room temperature. Cells were then incubated in primary antibodies diluted in blocking solution at room temperature overnight. After rinsing 3 times with PBS, cells were incubated with Alexa 488 or Alexa 594 conjugated secondary antibody made in goat (Life Technologies), diluted 1:200 in blocking solution for 2h at room temperature. Nucleus was stained with 4', 6-diamidino-2-phenylindole (DAPI, Life Technologies) and then rinsing 3 times with PBS. Cover slips with stained cells were mounted on glass slides with mounting medium. The primary antibodies used were the following: anti-glial fibrillary acidic protein (GFAP) mouse (1:500,); anti-Iba1 rabbit (1:500,); anti-CD11b rat (1:100,); anti-TDP43 rabbit polyclonal antibody (1:200, ProteinTech) and anti-TDP-43 mouse monoclonal (1:100, Abnova). Cells were viewed using a 40X objective on a DM5000B microscope (Leica). Statistical analyses were performed with GraphPad Prism 5.

## Results

### No change in mRNA expression for TDP-43 due to LPS treatment

**Fig 1:**
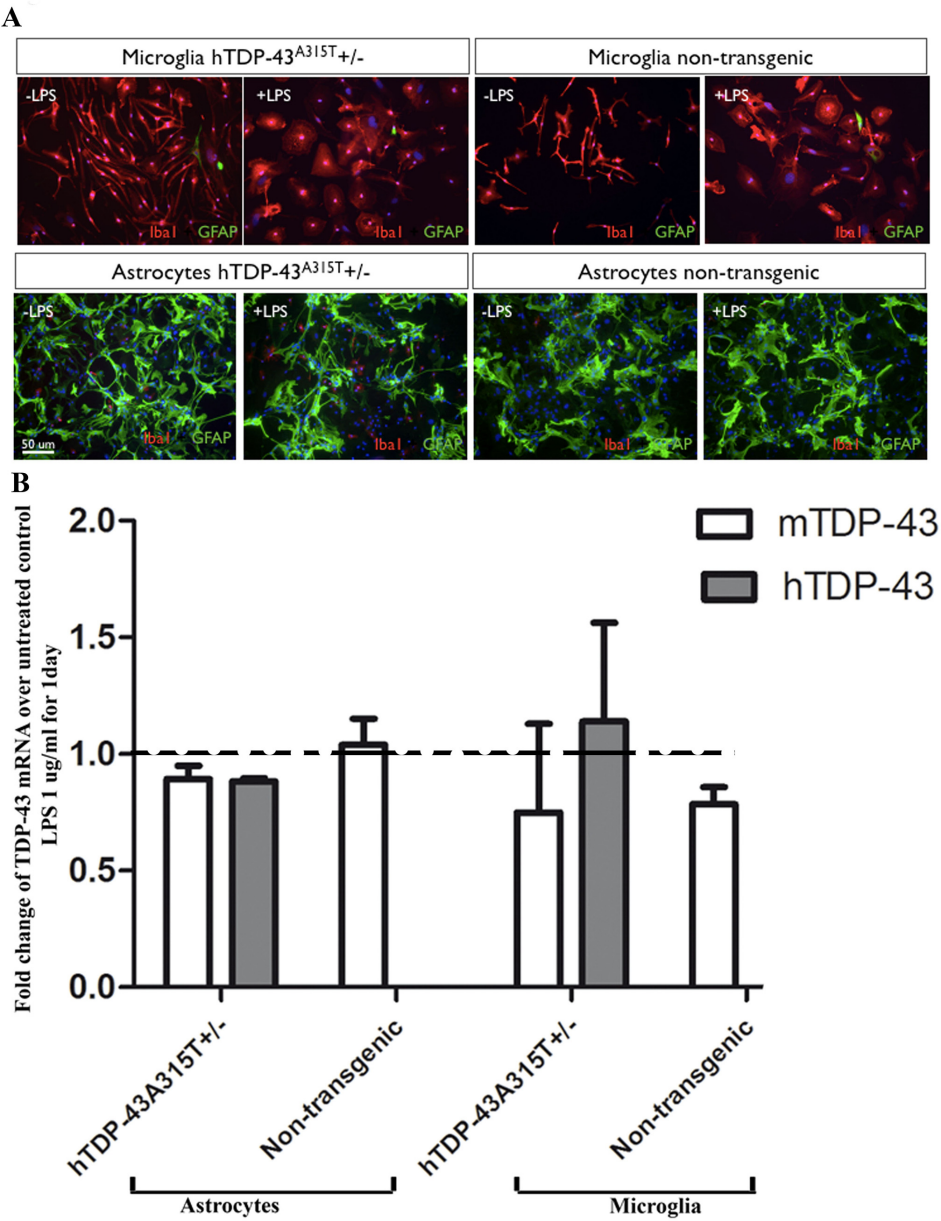
No change in mRNA expression for TDP-43 species due to LPS treatment. (A) Representative images of primary astrocytes and microglia cultures double stained for Iba 1(in red, marker of microglia) and GFAP (in green, marker of astrocytes). Nuclei of all cells were stained with DAPI (in blue). Using this double immunostaining we confirmed that we achieved a good separation of astrocytes from microglia, and resulting cultures were ~ 90% pure in one of the cell types. (B) Total RNA was extracted from LPS-treated (1ug/ml LPS for 1 day) and untreated cultures of astrocytes and microglia. Samples of total RNA were then subjected to real-time quantitative RT-PCR for human TDP-43 (hTDP-43, transgene) and mouse TDP-43 (mTDP-43, endogenous gene). Number of copies of hTDP-43 and mTDP-43 were normalized with the house-keeping gene Atp5 mRNA, in order to take in account variations in samples size. Levels of RNA in the LPS-treated sample were divided by the levels of mRNA in the respective untreated control, in order to get the fold changes in the expression with the LPS-treatment. Fold change equal to 1 indicate no effect of LPS in the expression of the gene, which was seen in most of the cultures analyzed for both mouse and human TDP-43.

Primary astrocytes and microglia were prepared from neonatal mice as described in Materials and Methods. As schematic representation of the approaches is shown in S1 Fig. Morphological changes of astrocytes and microglia to LPS treatment confirmed the activation of those cells. As expected, microglia from C57Bl6 (non-transgenic) mice and from hTDP-43^A315T^ transgenic mice went from a ‘resting’ form with long branching processes and small cellular body, in the absence of LPS, to a large ameboid form when treated with LPS (Fig 1A). LPS treatment of astrocytes or microglia did not cause significant changes in mRNA levels for the endogenous TDP-43 or human TDP-43 mutant, as detected by real-time quantitative RT-PCR (Fig 1B).

### Higher levels of TDP-43 protein in LPS-activated astrocytes and microglia

**Fig 2:**
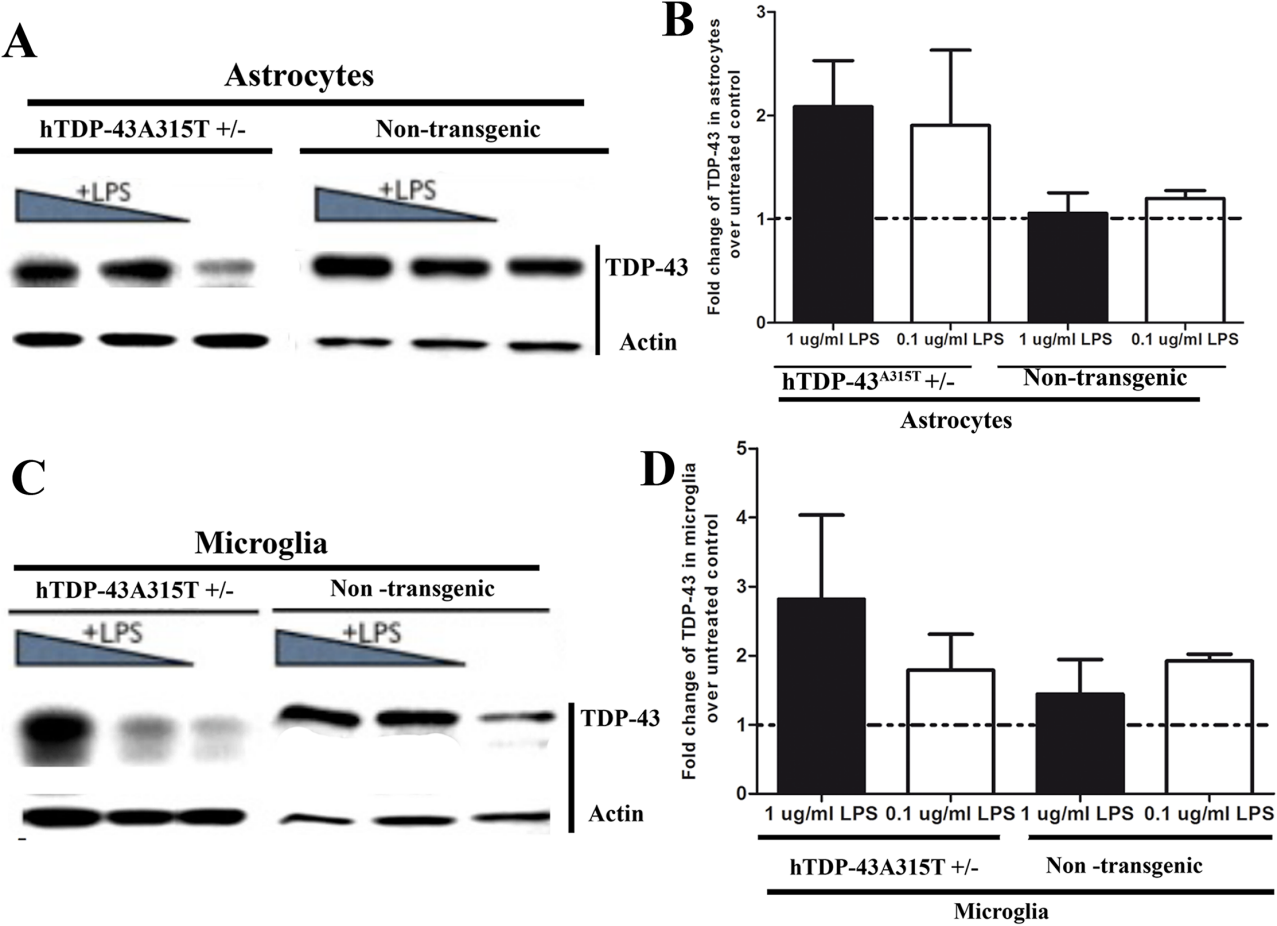
Higher levels of TDP-43 protein in LPS-activated astrocytes and microglia. Cultured astrocytes and microglia from transgenic (hTDP-43^A315T^) and non-transgenic litters were treated for 1 day with LPS at 1 μg/ml or 0.1 μg/ml. Total protein was extracted from LPS -treated and untreated cultures and analysed by immunoblotting. TDP-43 was detected using the polyclonal antibody (Proteintech # 10782-AP) which reacts with both human (transgenic) and mouse (endogenous) proteins. TDP-43 bands were normalized against actin to take into account the difference in protein loading. The exposure times are actually different between transgenic and non-transgenic blots. Due to higher quantities of TDP-43 (human plus mouse) in the transgenic cell cultures the exposure time was only 30 seconds whereas for the non-transgenic cultures it was 4 minutes. Fold change is the ratio of band intensity in LPS-treated cultures over the band intensity in the respective untreated control. (A) Representative western blot of TDP-43 from LPS treated or untreated astrocyte culture. (B) Quantitative analysis of western blot showed that levels of total TDP-43 was higher in LPS treated transgenic astrocyte culture than non-transgenic culture. (C) Representative western blot of TDP-43 from LPS treated or untreated microglia culture. (D) Quantitative analysis of western blot showed that levels of total TDP-43 were also higher in LPS-treated microglia from transgenic TDP-43^A315T^ mice than from C57BL6 mice.

Although the TDP-43 mRNA levels were not altered by LPS treatment, immunoblotting revealed higher amounts of TDP-43 protein from astrocytes and microglia when treated with LPS (Fig 2A–2D). We carried out the immunoblots with a widely used polyclonal antibody against TDP-43 [4,5, 8, 6,7, 29, 23], which reacts with both mouse and human TDP-43 species and yields a band at 43 kDa corresponding to the unmodified TDP-43.

### Accumulation of TDP-43 protein in the cytoplasm of LPS-activated astrocytes and microglia

**Fig 3:**
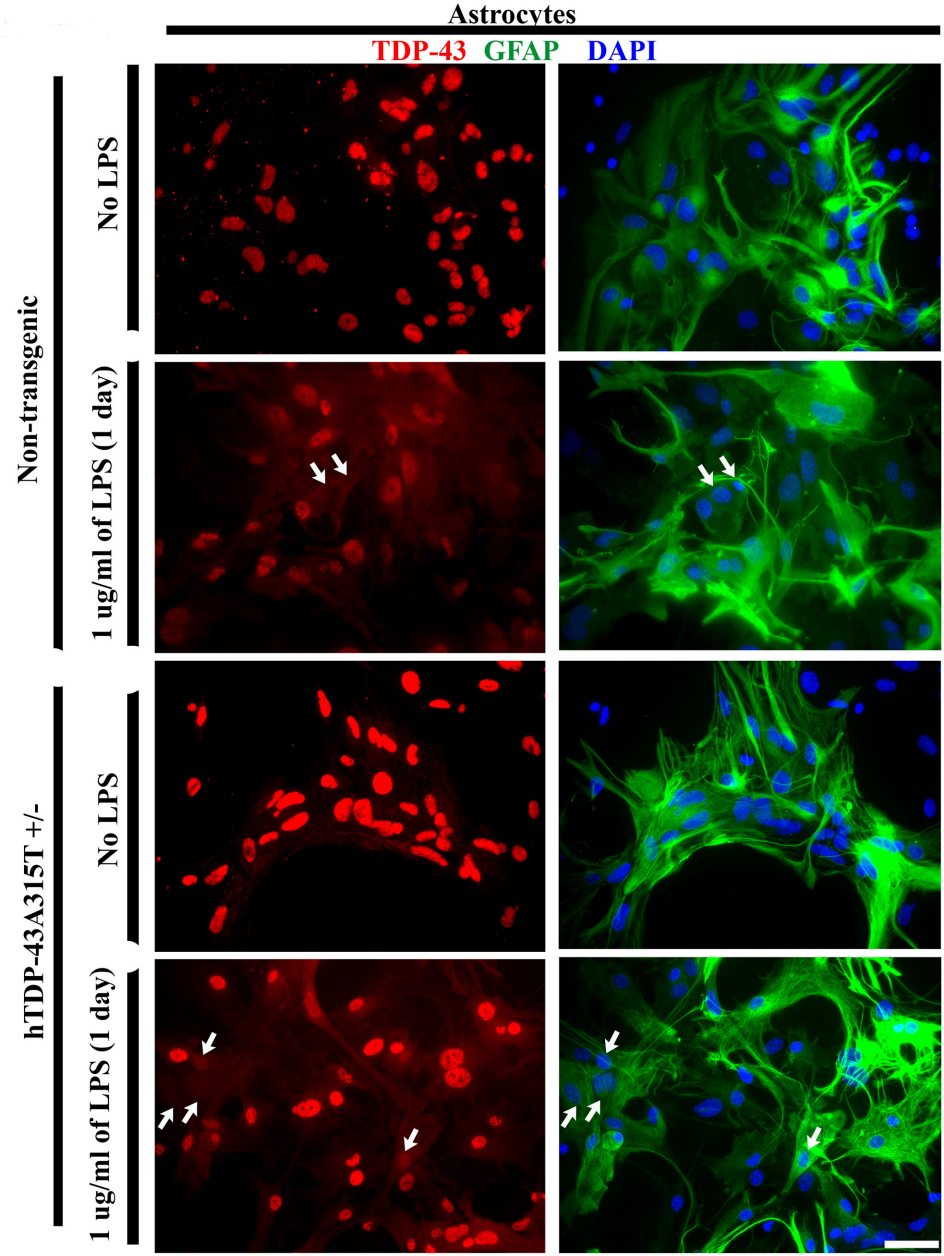
Cytoplasmic increase of TDP-43 in LPS-activated astrocytes. Representative images of astrocytes from hTDP-43^A315T^ transgenic and non-transgenic littermates, treated or not treated with LPS (1 μg/ml) for one day. Astrocytes are double stained for TDP-43 (red) and GFAP (green) while nuclei are stained with DAPI (blue). Immunofluorescence for TDP-43 increased in the cytoplasm of LPS-treated astrocytes in both non transgenic (2^nd^ panel) and transgenic culture (4^th^ panel) as compared to the untreated control (1^st^ and 3^rd^ panel). However, the overall cytoplasmic increase of TDP-43 was more pronounced in LPS-treated astrocytes from hTDP-43^A315T^ transgenic mice than in LPS-treated astrocytes from C57Bl6 mice. Arrows point on cells presenting a decrease in nuclear TDP-43. Scale bar 100μm.

**Fig 4:**
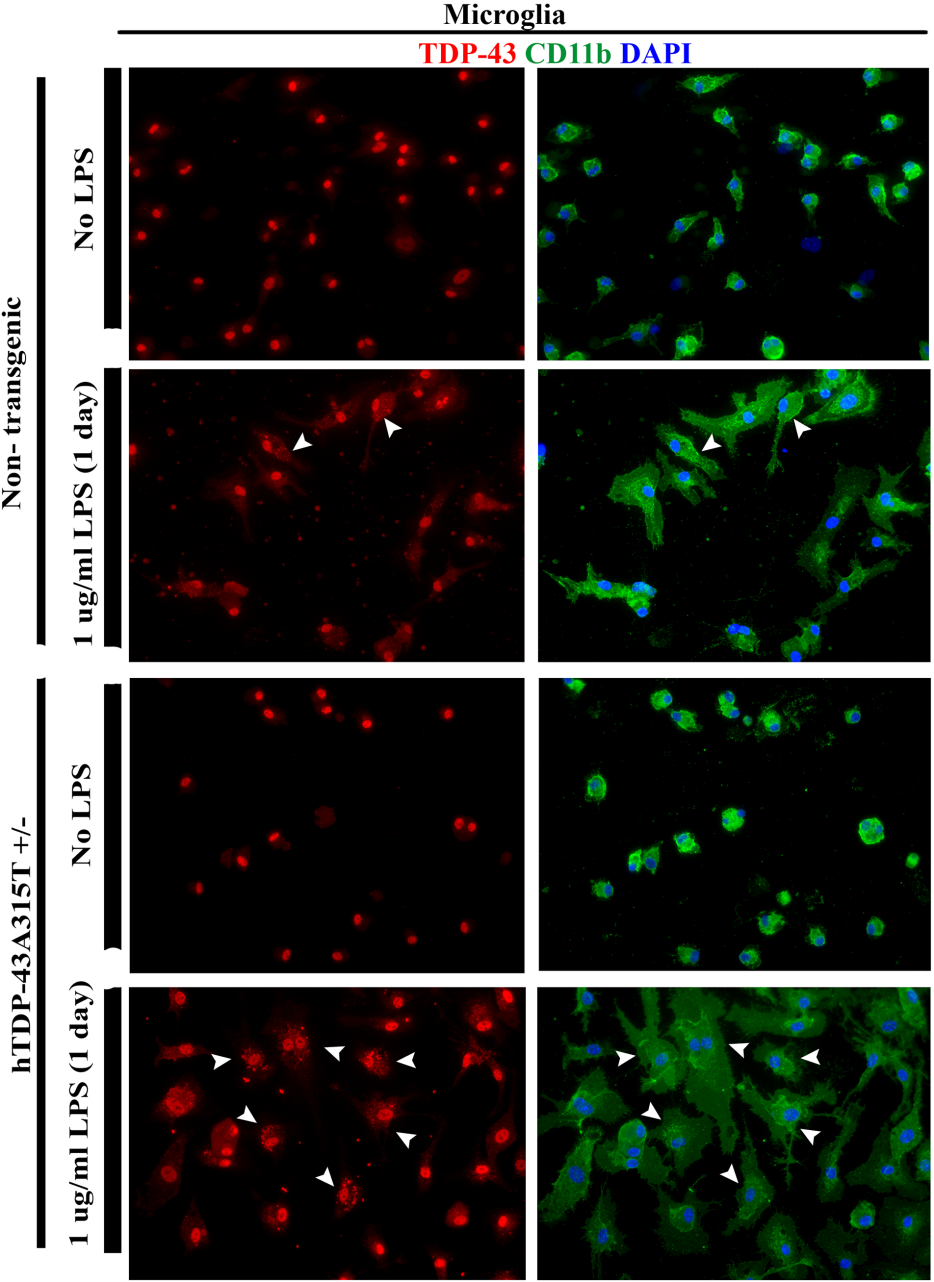
Cytoplasmic aggregates of TDP-43 in LPS-activated microglia. Representative images of microglia from hTDP-43^A315T^ transgenic and control littermates, treated or not treated with LPS (1μg/ml) for one day. Microglial cultures were double stained for polyclonal TDP-43 (red) and CD11b (green) whereas the nuclei were stained with DAPI (blue). No cytoplasmic aggregates of TDP-43 were found in untreated microglia from control C57Bl6 mice (1^st^ panel) or from hTDP-43^A315T^ transgenic mice (3^rd^ panel). Treatment with LPS resulted in formation of small cytoplasmic TDP-43 aggregates in microglia from control C57Bl6 mice (2^nd^ panel) and from hTDP-43^A315T^ transgenic mice (4^th^ panel). However, TDP-43 punctate aggregates were more abundant and of higher intense staining in LPS-treated microglia (4^th^ panel) from hTDP-43^A315T^ transgenic when compared to microglia from C57Bl6 mice (2^nd^ panel). Arrowheads point to cells with cytoplasmic aggregates of TDP-43.

To test the effect of LPS on TDP-43 distribution in cytoplasm and nucleus of glial cells, primary glial cultures from non-transgenic and transgenic hTDP-43^A315T^ mice were treated with 1μg/ml of LPS for 1 day. The sub-cellular localization of TDP-43 in astrocytes and microglia was analyzed by immunocytochemistry. Cell cultures were double stained for TDP-43 and GFAP (astrocyte marker) (Fig 3) or CD11b (microglia marker) (Fig 4).

As shown in Fig 3, LPS treatment of non-transgenic and hTDP-43^A315T^ transgenic astrocytes resulted in redistribution of TDP-43 from nucleus to cytoplasm. As observed in Fig 4, most LPS-treated microglia also presented altered cytoplasmic distribution of TDP-43 with punctate aggregates of TDP-43, resembling those described in samples from patients with ALS and FTLD [4, 7]. Although some TDP-43 positive cytoplasmic punctate structures were also observed in LPS-treated microglial cells from non-transgenic mice, the punctate staining was less intense than in LPS-treated microglial cells from transgenic hTDP-43^A315T^ mice.

**Fig 5:**
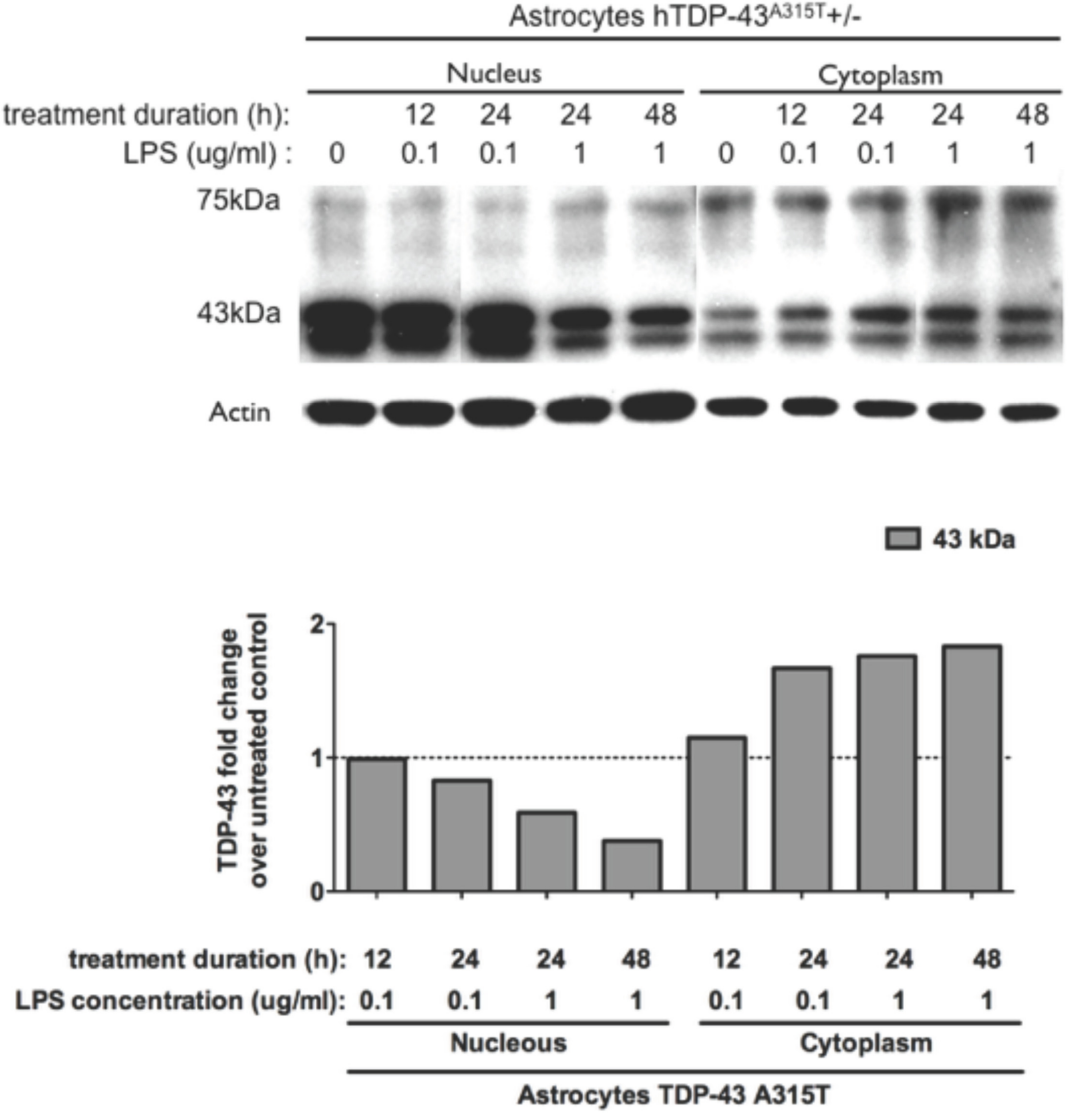
Translocation of TDP-43 from the nucleus to the cytoplasm in astrocytes and microglia with LPS-treatment. (A) Nuclear and cytoplasmic protein fractions of astrocytes were analyzed by Western blot. The intensity of the 43kDa TDP-43 bands was divided by the intensity of the respective actin band to take in account the differences in the protein loading. Fold change is the ratio of the 43 kDa band intensity in the LPS-treated cultures over the band intensity in the respective untreated controls, after normalized by the intensity of the correspondent actin bands.

Subcellular fractionation of astrocyte cultures was performed. Nuclear and cytoplasmic fractions were analyzed by Western blot (Fig 5). TDP-43 was detected using a polyclonal antibody (Proteintech #10782-2-AP) which reacts with both human (transgenic) and mouse (endogenous) TDP-43. The intensity of TDP-43 bands was divided by the intensity of the respective actin band to take in account the differences in the protein loading. As shown in Fig 5, the treatment with LPS resulted in loss of TDP-43 protein in the nuclear fraction and in increased levels of TDP-43 in the cytoplasmic fraction.

**Fig 6:**
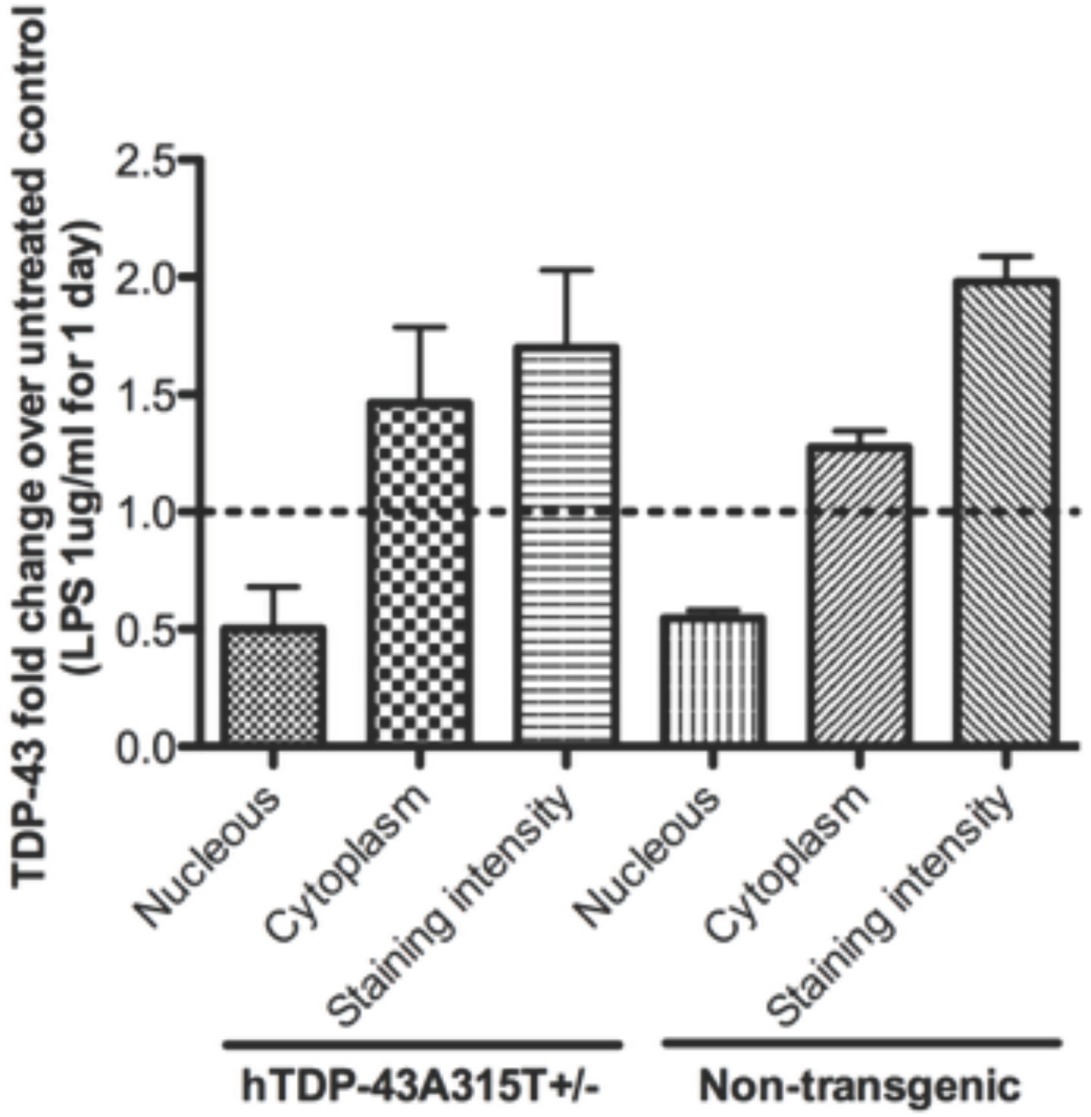
LPS treatment caused changes in nuclear and cytosolic TDP-43 immunostaining intensities in microglia cultures. The staining intensities were measured using the software Imaris on images taken with the confocal microscope Olympus IX81 and the software Olympus Fluoview ver 3.1a. More than 50 cells were analysed per cultures, 3 cultures of transgenic microglia and 3 cultures of non-transgenic microglia (N = 3). Fold changes were calculated by dividing the average intensities in the LPS-treated cultures over the average intensities in the untreated cultures. Graphic bars represent average and standard error of the mean values calculated with software GraphPad Prism 5.

As shown in Fig 6, the nuclear and cytosolic TDP-43 staining intensities were measured in microglial cultures using the software Imaris on images taken with the confocal microscope. The results show that LPS treatment caused reduction in nuclear immunostaining of TDP-43 and increased cytoplasmic immunostaining of TDP-43.

### Treatment with TNF-α increases cytoplasmic TDP-43 in NSC-34 cells

**Fig 7:**
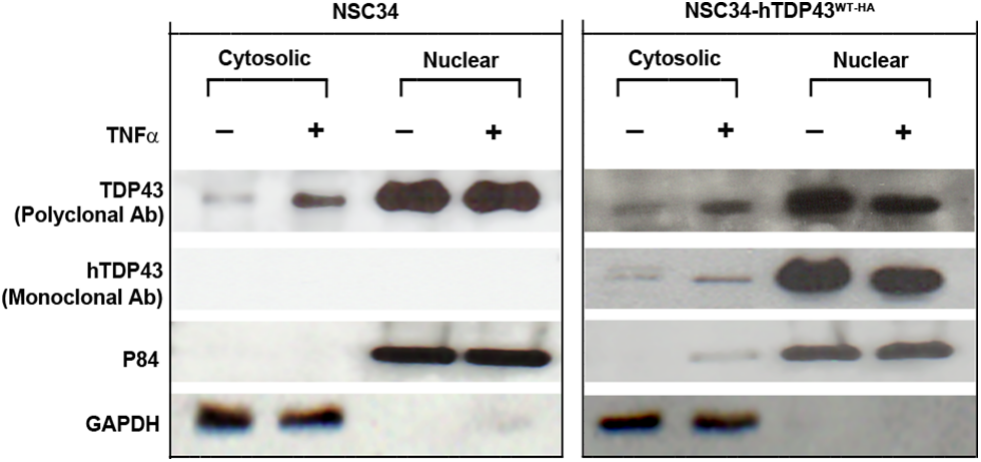
TNF-α increases cytoplasmic localization of TDP-43 in NSC-34 cells. Non-transfected NSC-34 cells and hTDP-43WT-HA stably-transfected NSC-34 cells were treated with recombinant TNF-α at a concentration of 10 ng/mL for 6h. Non-TNF treated cells served as controls. Following treatment, the subcellular distribution of TDP-43 was determined by Western blot analysis. Immunoblotting with polyclonal TDP-43 antibody showed increased cytoplasmic TDP-43 in TNF-α-treated NSC-34 cells (left first panel) and hTDP-43WT-HA stably transfected cells (right first panel). Similarly, immunoblotting with monoclonal TDP-43 antibody (specific for human TDP-43) showed increased levels of cytoplasmic TDP-43 in TNF-α-treated hTDP-43WT-HA stably-transfected NSC-34 cells (2nd left panel). Immunoblots were reprobed with P84 antibody (nuclear marker) and GAPDH antibody (cytoplasmic marker).

The cellular/molecular events that may lead to abnormal neuronal distribution of TDP-43 in neuronal cells are largely unknown. We investigated whether inflammatory stimuli could promote mislocalization of TDP-43 from nucleus to cytoplasm in motor neuron-like NSC-34 cell lines. Non-transfected NSC-34 cells and NSC-34 cells stably transfected with hTDP-43WT-HA were treated with recombinant mouse TNF-α at a concentration of 10 ng/mL for 6h. Non-TNF treated cells served as controls. Following treatment, the subcellular distribution of TDP-43 was determined by Western blot analysis complemented by immunocytochemistry. As expected, the majority of TDP-43 was located in nucleus. However, TNF-α treatment induced cytoplasmic mislocalization of TDP43 in non-transfected cells as well as in cells stably transfected with hTDP-43WT-HA (Fig 7). Thus, the results suggest that the activation of TNF-α/NF-κB signaling pathway can induce abnormal cytoplasmic mislocalization and aggregation of TDP-43 in neuronal cells.

### Chronic LPS administration exacerbated cytoplasmic mislocalization and aggregation of TDP-43 in hTDP-43^A315T^ transgenic mice

**Fig 8:**
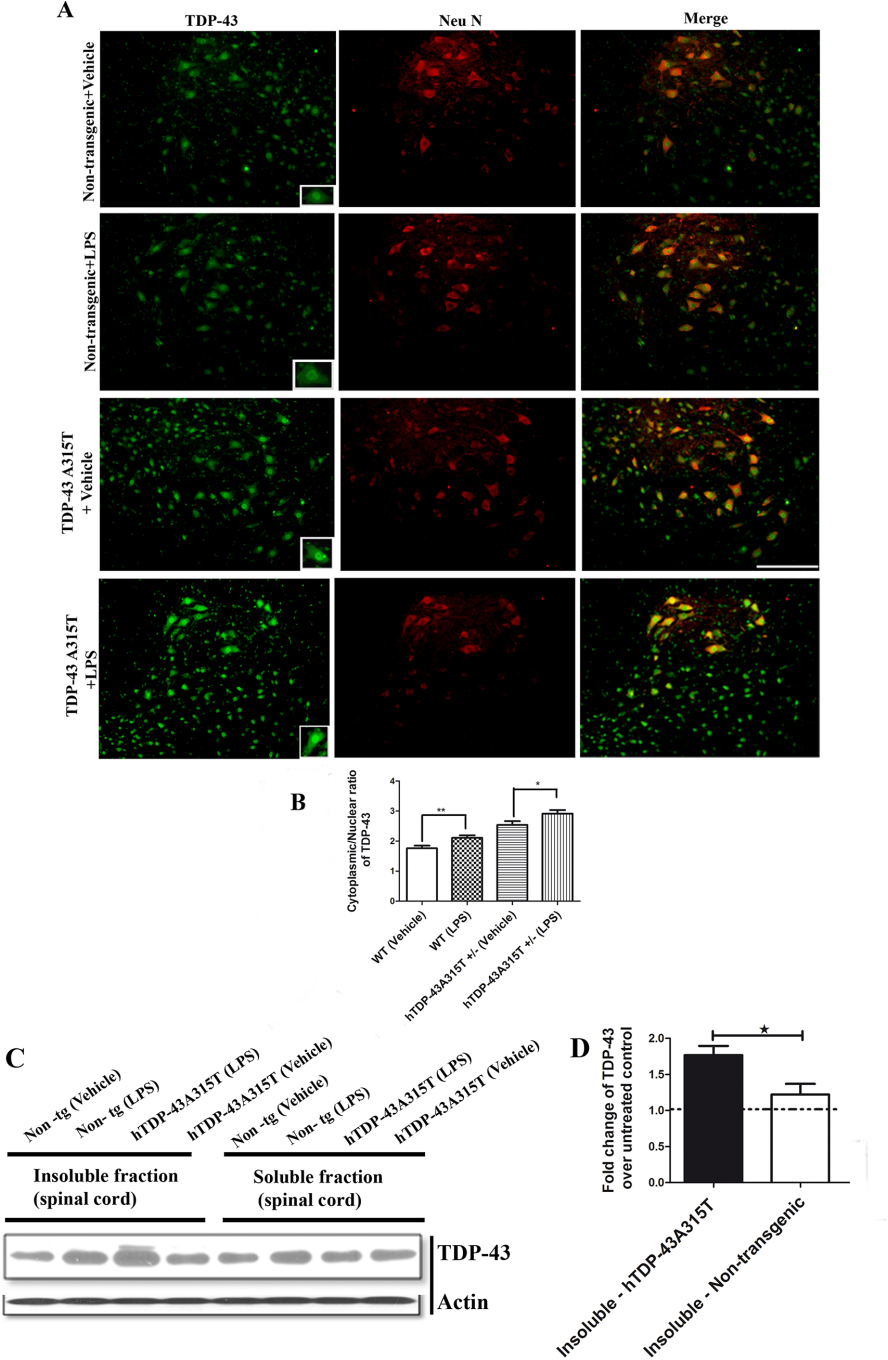
Chronic LPS treatment exacerbated cytoplasmic mislocalization and aggregation of TDP-43 in hTDP-43^A315T^ transgenic mice. Non-transgenic and transgenic mice expressing hTDP-43^A315T^ mutant at 6 months of age were i.p. injected with LPS (1mg/kg) or vehicle solution once per week up to 2 months. (A) Representative images of spinal cord sections of non-transgenic and hTDP-43^A315T^ transgenic mice, vehicle and LPS treated were double stained with polyclonal TDP-43 (green) and Neu N (red). (B) Measurement of cytoplasmic to nuclear ratio of TDP-43 staining showed increased ratio in LPS treated transgenic as well as non-transgenic mice (N = 4 per group, 8 sections of spinal cord from 4 different animals for each group, *<0.05, **<0.001 by student’s t test.) (C) Chronic treatment of hTDP-43^A315T^ transgenic mice with LPS enhanced levels of insoluble TDP-43 in spinal cord. Total protein was extracted from spinal cords of LPS or vehicle-treated mice and sub-fractionated into insoluble and soluble fractions. Sub-fractionated samples were then analyzed by Western blot using the polyclonal TDP-43 antibody. (D) The intensity of TDP-43 bands were divided by the intensity of the respective actin band to take in account the differences in the protein loading and then the fold change was calculated. The fold change is the ratio of the band intensity in the LPS-treated mice over the band intensity in the respective vehicle-treated controls. Fold changes are all higher than 1, indicating that LPS treatment led to increased levels of TDP-43 protein. Groups were compared using t-test. *p value = 0.03 by student’s t test (N = 4 per group, spinal cord from 4 different animals were used to extract protein for each group). Scale bar 50μm.

To address whether LPS treatment may also induce TDP-43 mislocalization and aggregation *in vivo*, 6 months old non-transgenic mice and hTDP-43^A315T^ transgenic mice were chronically i.p. injected once a week with LPS (1 mg/kg) or vehicle solution starting at 6 months of age for a duration of 2 months. After two months of LPS treatment, the spinal cords were collected and examined by immunostaining or western blotting for TDP-43 immunodetection. Spinal cord sections double immunostained with polyclonal antibody for TDP-43 and neuronal marker (Neu N) showed increased cytoplasmic immunostaining in motor neurons of transgenic mice treated with LPS (Fig 8A). Measurement of cytoplasmic and nuclear fluorescence of TDP-43 in spinal cord neurons of transgenic mice showed an increased cytoplasmic to nuclear ratio of TDP-43 intensity in LPS-treated mice as compared to the vehicle-treated mice (Fig 8B).

In ALS, TDP-43 does not remain in its normal nuclear location, but instead forms insoluble aggregates in both the nucleus and cytoplasm of affected neurons [4, 7]. In order to analyze the presence of insoluble TDP-43 aggregates after LPS treatment, soluble and insoluble protein fractions in spinal cord lysates were prepared as described earlier [31]. No change was detected in the amount of soluble TDP-43 in the spinal cord after chronic LPS treatment of non-transgenic and hTDP-43^A315T^ transgenic mice (Fold change ~1) (Fig 8C and 8D). In contrast, levels of insoluble TDP-43 were increased after chronic LPS treatment in the spinal cord of non-transgenic and hTDP-43^A315T^ transgenic mice (Fold change > 1) (Fig 8C and 8D). The effect of LPS treatment on TDP-43 aggregation was more pronounced in the hTDP-43^A315T^ transgenic mice when compared to non-transgenic mice (Fig 8D).

**Fig 9:**
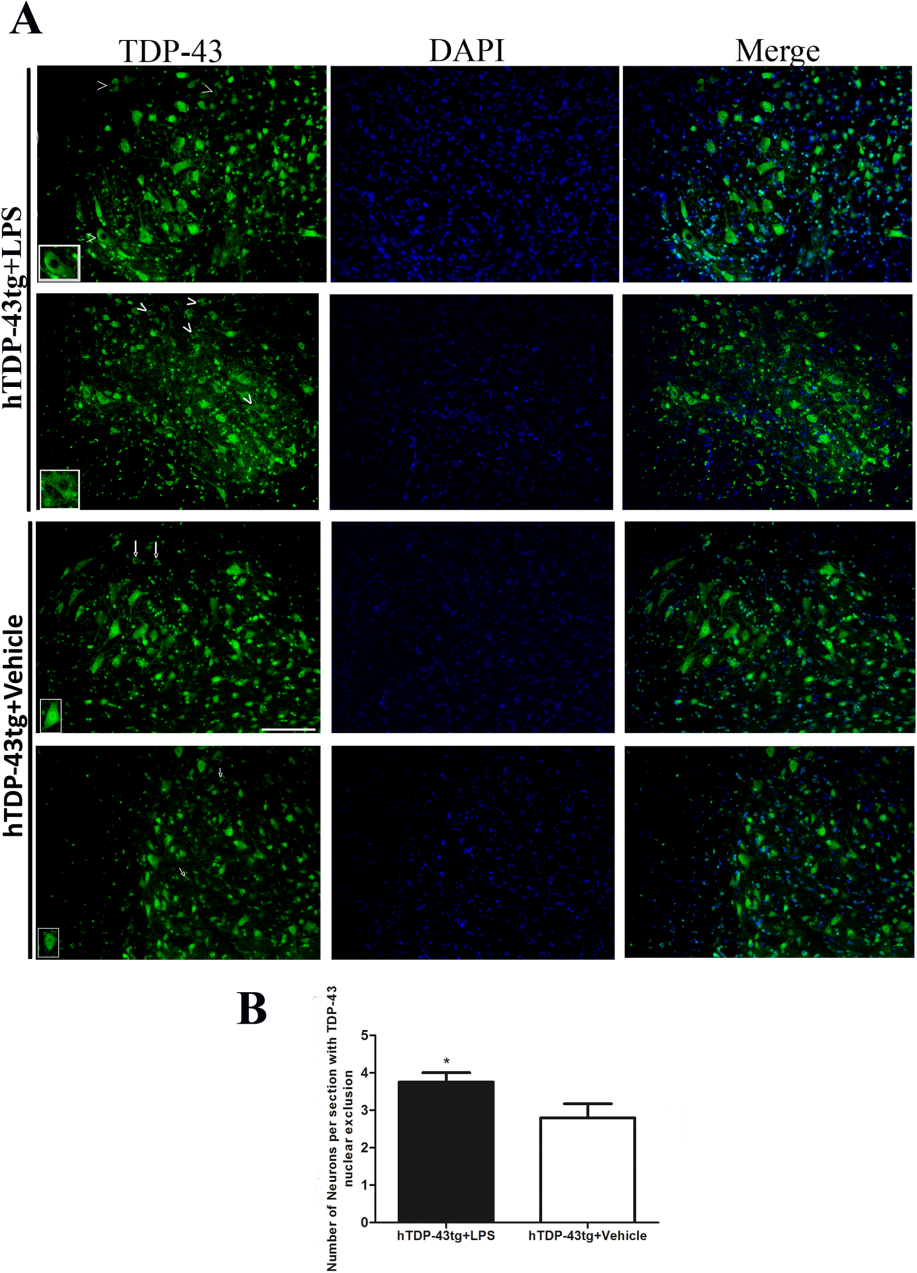
Chronic LPS treatment enhanced nuclear exclusion of TDP-43 in spinal neurons of hTDP-43^A315T^ transgenic mice. Transgenic mice expressing hTDP-43^A315T^ mutant at 6 months of age were i.p. injected with LPS (1mg/kg) or vehicle solution once per week for a period of 2 months. (A) Spinal cord sections from LPS and vehicle-treated mice were immunostained with monoclonal antibody specific for human TDP-43. As revealed by immunostaining, more neuronal cells in LPS-treated spinal cord sections exhibited loss of nuclear TDP-43 staining (first and second panels) than vehicle (third and fourth panels). Insets in the first and second panels show magnified motor neurons of LPS-treated mice with nuclear loss of TDP-43. Arrow heads in first and second panels show neurons exhibiting nuclear loss of TDP-43 in LPS-treated hTDP-43^A315T^ tg mice while arrows in third and fourth panel show neurons exhibiting nuclear loss of TDP-43 in vehicle-treated hTDP-43^A315T^ tg mice. All representative sections are from different mice for each treatment group. DAPI was used to stain nuclei. Scale bar = 50μm. (B) Number of spinal neurons per section exhibiting nuclear loss of TDP-43 was higher in LPS-treated mice as compared to vehicle-treated mice (*p* = 0.043 by student’s t test, N = 4, 6 spinal cord sections from 4 different animals at 8 months were analyzed for each group).

To further confirm the effect of LPS treatment on abnormal distribution of TDP-43, spinal cord sections from LPS and vehicle-treated mice were immunostained with monoclonal antibody specific for human TDP-43. As revealed by immunostaining, spinal neurons from hTDP-43^A315T^ transgenic mice exhibited more cytoplasmic TDP-43 immunostaining when injected with LPS rather than vehicle. LPS treatment increased the number of neuronal cells with nuclear depletion of TDP-43 and with cytoplasmic TDP-43 aggregates (Fig 9). These *in vivo* results are in agreement with our findings with *in vitro* cell cultures that activation of NF-κB pathway by inflammatory stimuli (LPS or TNF-α) can promote cytoplasmic mislocalization and aggregation of TDP-43.

## Discussion

The results presented here demonstrate that inflammatory stimuli such as TNF-α or LPS can promote cytoplasmic mislocalization and aggregation of TDP-43 in glial and neuronal cells, a proteinopathy similar to what has been observed in ALS cases [7]. Some factors are known to trigger TDP-43 redistribution from the nucleus to cytoplasm with formation of protein aggregates including axotomy, cell stressors, over-expression and mutations of TDP-43 gene [21, 17]. This is the first report of inflammation being a factor that can contribute to TDP-43 proteinopathy.

In this study, we took advantage of transgenic mice bearing a genomic fragment encoding human mutant TDP-43^A315T^ linked to familial ALS [11–12]. This transgenic mouse model was previously characterized [23,29]. Unlike other transgenic mice overexpressing TDP-43 species under the control of strong neuronal gene promoters [32–35], the hTDP-43^A315T^ mice overexpress at moderate levels the TDP-43 transgene ubiquitously under its own promoter. Thus, it was possible to derive from the hTDP-43^A315T^ transgenic mice primary cultures of astrocyte and microglia that expressed TDP-43^A315T^ mutant. The LPS treatment increased the total amount of TDP-43 protein in both microglia and astrocyte cultures (Fig 2), but without corresponding increases at mRNA levels (Fig 1). Moreover, LPS treatment of microglia and astrocytes enhanced the cytoplasmic mislocalization TDP-43. In microglia, LPS exposure also led to formation of cytoplasmic TDP-43 punctate aggregates (Fig 4).

Furthermore, *in vivo* evidence for an involvement of inflammation in TDP-43 pathology was provided by the chronic LPS administration in hTDP-43^A315T^ transgenic mice starting at 6 months of age. These transgenic mice exhibit during aging cognitive and motor impairments, as well as progressive formation of TDP-43 cytoplasmic aggregates characteristic of ALS [29]. After two months of LPS treatment, the hTDP-43^A315T^ mice exhibited higher levels of insoluble TDP-43 than vehicle-treated mice (Fig 8C and 8D). In addition, measurement of TDP-43 immunostaining intensity in spinal motor neurons revealed that LPS treatment increased the cytoplasmic to nuclear ratio of TDP-43 immunostaining (Fig 8A and 8B). Immunofluorescence microscopy of spinal cord sections with monoclonal antibody against human TDP-43 showed that LPS-treatment resulted in nuclear TDP-43 depletion and in cytoplasmic TDP-43 aggregates (Fig 9). There is evidence that such abnormal cytosolic TDP-43 aggregates can be toxic [36, 37].

In summary, the *in vitro* and *in vivo* results presented here suggest that inflammation, induced by stimuli of NF-κB signaling such as TNF-α or LPS, may be a mediator of TDP-43 proteinopathy which constitutes a pathological hallmark of ALS and FTLD [4–9]. Chronic immune activation is common feature of neurodegenerative disorders including ALS. The sources of inflammation in ALS remain to be defined. Somehow, ALS patients have increased levels of LPS in the blood as well as an up-regulation of LPS/TLR-4 signaling associated genes in the peripheral blood monocytes [27–28]. An upregulation of TDP-43 levels detected in ALS is a phenomenon that may also contribute to enhance the NF-κB response to inflammatory stimuli [23]. In diseases, neurons may be damaged by a side-effect of inflammation and pro-inflammatory cytokines. Of particular interest is our finding that TNF-α, a cytokine produced by activated microglial cells, may activate the NF-κB pathway in motor neurons to promote cytoplasmic mislocalization and aggregation of TDP-43 (Fig 7). Apart from the possibility that innate immune activation of microglial cells can damage neurons and aggravate neuronal TDP-43 proteinopathy via TNF-α release, it is remarkable that treatment with LPS of cultured microglial cells, even from normal mice, triggered abnormal cytoplasmic aggregation of TDP-43. This raises the possibility that activated microglia might also constitute a source of insoluble TDP-43 aggregates for the seeding and prion-like propagation of TDP-43 aggregates.

## Supporting Information

S1 Fig Schematic representation of the approaches for in vitro (A) and in vivo studies (B). **(A)** Primary astroglia and microglia cultures were prepared from brain tissues of neonatal mice and then subjected to LPS treatment as described in Materials and Methods. **(B)** To trigger a systemic innate immune response in the CNS, presymptomatic 6-month-old hTDP-43^A315T^ mice and their non-transgenic (wild-type) littermates received intraperitoneal i.p. injection of LPS (1 mg/kg of body weight) diluted in 100 μl of saline. Mice were i.p. injected once a week for duration of two months.
(PDF)
