# Supplementary figures and images for: Inflammation Induces TDP-43 Mislocalization and Aggregation
Source: PLoS One. 2015 Oct 7;10(10):e0140248. doi: 10.1371/journal.pone.0140248 (PMC4596857; doi:10.1371/journal.pone.0140248)

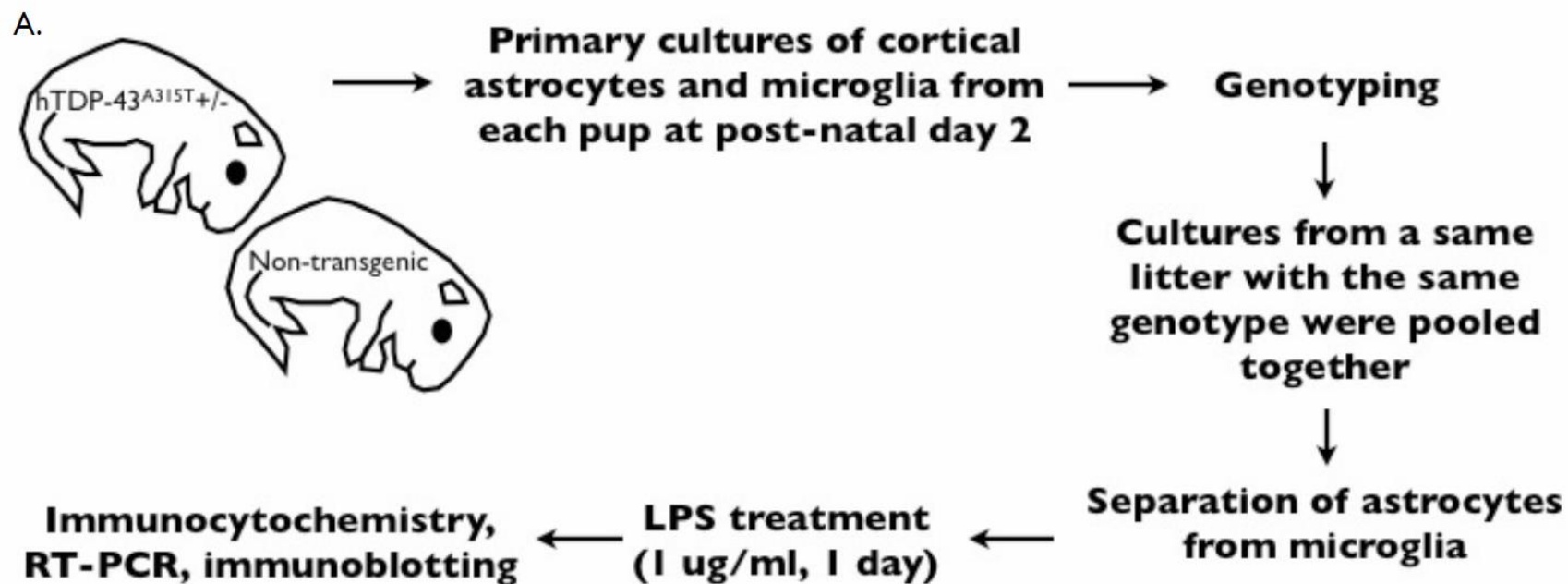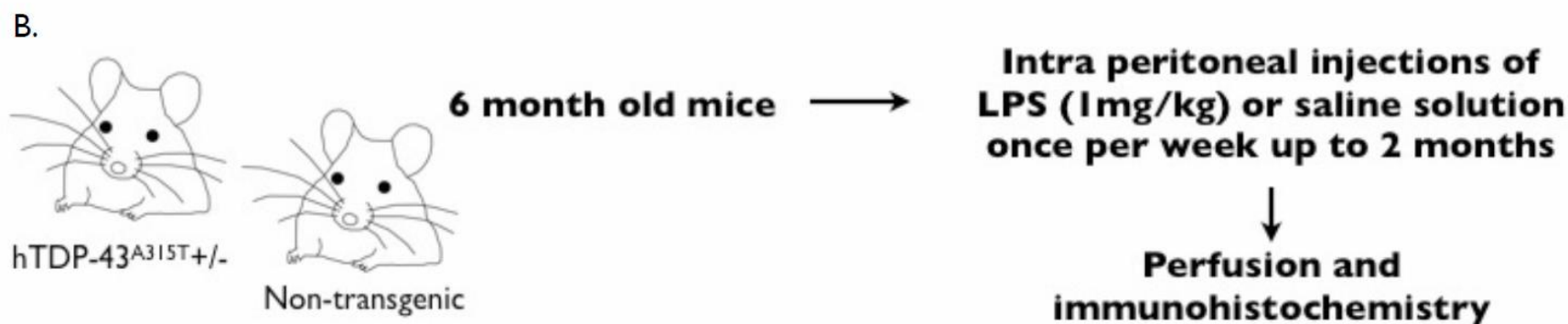

Supplement: S1 Fig — (A) Primary astroglia and microglia cultures were prepared from brain tissues of neonatal mice and then subjected to LPS treatment as described in Materials and Methods. (B) To trigger a systemic innate immune response in the CNS, presymptomatic 6-month-old hTDP-43A315T mice and their non-transgenic (wild-type) littermates received intraperitoneal i.p. injection of LPS (1 mg/kg of body weight) diluted in 100 μl of saline. Mice were i.p. injected once a week for duration of two months. (PDF) [file pone.0140248.s001.pdf]
